# Effects of preoperative magnetic resonance image on survival rates and surgical planning in breast cancer conservative surgery: randomized controlled trial (BREAST-MRI trial)

**DOI:** 10.1007/s10549-023-06884-5

**Published:** 2023-02-14

**Authors:** Bruna Salani Mota, Yedda Nunes Reis, Nestor de Barros, Natália Pereira Cardoso, Rosa Maria Salani Mota, Carlos Shimizu, Tatiana Cardoso de Mello Tucunduva, Vera Christina Camargo de Siqueira Ferreira, Rodrigo Goncalves, Maíra Teixeira Doria, Marcos Desidério Ricci, Angela Francisca Trinconi, Cristina Pires Camargo, Rachel Riera, Edmund Chada Baracat, José Maria Soares Jr, José Roberto Filassi

**Affiliations:** 1 Setor de Mastologia da Disciplina de Ginecologia do Departamento de Obstetricia e Ginecologia, Hospital das Clínicas, Faculdade de Medicina da Universidade de São Paulo (FMUSP), Av. Dr. Arnaldo, 251; 4o andar Secretária Cirúrgica, São Paulo, SP 01246-000 Brazil; 2 Hospital Nossa Senhora das Graças, Curitiba, PR Brazil; 3 Universidade Federal do Ceará, Fortaleza, CE Brazil; 4 Microsurgery and Plastic Surgery Laboratory, School of Medicine, Universidade de São Paulo, São Paulo, Brazil; 5 Hospital Sírio Libanês, São Paulo, Brazil

**Keywords:** Magnetic resonance imaging, Breast cancer, Conservative breast cancer surgery, Randomized controlled trial

## Abstract

**Background:**

Breast magnetic resonance imaging (MRI) has high sensitivity in detecting invasive neoplasms. Controversy remains about its impact on the preoperative staging of breast cancer surgery. This study evaluated survival and surgical outcomes of preoperative MRI in conservative breast cancer surgery.

**Methods:**

A phase III, randomized, open-label, single-center trial including female breast cancer participants, stage 0–III disease, and eligible for breast-conserving surgery. We compared the role of including MRI in preoperative evaluation versus radiologic exam routine with mammography and ultrasound in breast cancer conservative candidates. The primary outcome was local relapse-free survival (LRFS), and secondary outcomes were overall survival (OS), mastectomy rate, and reoperation rate.

**Results:**

524 were randomized to preoperative MRI group (*n* = 257) or control group (*n* = 267). The survival analysis showed a 5.9-years LRFS of 99.2% in MRI group versus 98.9% in control group (HR = 0.72; 95% CI 0.12—4.28; *p* = 0.7) and an OS of 95.3% in the MRI group versus 96.3% in the control group (HR = 1.37 95% CI 0.59–3.19; *p* = 0.8). Surgical management changed in 21 ipsilateral breasts in the MRI group; 21 (8.3%) had mastectomies versus one in the control group. No difference was found in reoperation rates, 22 (8.7%) in the MRI group versus 23 (8.7%) in the control group (RR = 1.002; 95% CI 0.57–1.75; *p* = 0.85).

**Conclusion:**

Preoperative MRI increased the mastectomy rates by 8%. The use of preoperative MRI did not influence local relapse-free survival, overall survival, or reoperation rates.

## Introduction

Conservative surgery is the current practice for early-stage breast cancer [1, 2]. The efficacy and safety of this procedure depend on the precise and accurate assessment of the extension of the disease and the achievement of clear margins [3]. Therefore, with clinical examination and mammography (associated with breast ultrasound in selected cases), preoperative evaluation is essential. This combined approach enhances accuracy and diminishes the surgeons' odds of positive margins on the surgical specimen [4, 5].

Since breast magnetic resonance imaging (MRI) has a high sensitivity (95–100%) to detect invasive neoplasms [6], its role in the preoperative planning of breast cancer surgery has been investigated. However, controversy remains as to whether preoperative staging with breast MRI might impact clinical and surgical outcomes [7]. In a recent review, including 19 studies, preoperative MRI was associated with increased mastectomy rates and did not yield statistically significant differences in re-excision or positive margins rates[8].

Investigating the potential benefits of preoperative breast MRI, either by finding true synchronous lesions (thus lessening reoperation rates), improving survival outcomes, or even reducing overall costs, might be a critical point for establishing its role in the healthcare of breast cancer patients.

In this scenario, we planned and conducted the BREAST-MRI Trial to determine whether preoperative breast MRI may impact in survival and surgical outcomes in selected patients.

## Methods

### Trial design and setting

BREAST-MRI is a phase III, randomized, open-label, single-center trial including female breast cancer participants with stage 0-III disease and eligible for breast-conserving surgery at Instituto do Câncer do Estado de São Paulo (ICESP, Brazil) from November 2014 to July 2020.

### Participants

Inclusion criteria were those women older than 18 with stage 0–III breast cancer, according to American Joint Committee on Cancer 7th Edition [9], who were candidates for breast-conserving surgery. Exclusion criteria were contraindication for MRI (i.e., metal implants, claustrophobia), neoadjuvant treatment, chronic renal failure on dialysis, personal history of breast cancer or other neoplasms, pregnancy or lactation in the last six months, mental illness and/or difficulties in comprehending the study, refusal to perform breast MRI during the trial or had undergone surgery in another hospital.

This trial was approved by the Local Ethics Committee (Approval Number 974.504) and registered in the Clinical Trials Database (NCT02798796).

### Interventions

After providing full informed consent, all eligible women were submitted to triple assessment breast evaluation which consists of clinical breast examination, bilateral mammogram, and ultrasound in the breast image center at ICESP, and then randomized to perform or not MRI for preoperative evaluation.

### Breast image

#### Mammogram

The mammogram was performed using a digital unit (Selenia, Hologic, Bedford, Mass) with the acquisition of at least two views (craniocaudal and mediolateral oblique) for each breast. The images were analyzed at a dedicated mammography workstation (Selenia, Hologic, Bedford, Mass). The Breast density on the mammogram was assessed using The American College of Radiology’s BI-RADS® fifth edition classification: A—breasts are almost entirely fat; B—there are scattered areas of fibro glandular density; C—breasts are heterogeneously dense; D—breasts are extremely dense.

#### Ultrasound

The ultrasonography was performed by a dedicated breast-imaging physician with a multi-frequency transducer (10–15 MHz, Logiq E9, General Electric Medical Systems, Milwaukee, Wisconsin). Each breast was scanned in two different planes, including the lymphatic drainage (axilla and internal thoracic).

#### Breast resonance

Bilateral and simultaneous breast MRI was performed using a 1.5 T magnet (Signa HDXT, General Electric Medical Systems, Milwaukee, Wisconsin). Images were obtained before and after a 0.1 mmol/kg bolus injection of intravenous gadolinium contrast with an infusion pump in the axial plane. The acquisition protocol included a pre-contrast fat-suppressed T2 weighted Fast Spin Echo with a slice thickness of 3 mm, a fat-suppressed T1 3D gradient-echo pre (one sequence) and post (3 sequences) contrast with a slice thickness of 1.2—1.5 mm and acquisition time < 90 s and subtracted images. Our protocol also included diffusion-weighted imaging with a b value at 0 and 800 s/mm2.

The MRI, ultrasound, and mammogram interpretation were performed by two radiologists with more than five years of experience in breast imaging.

#### Surgical management

**Fig. 1:**
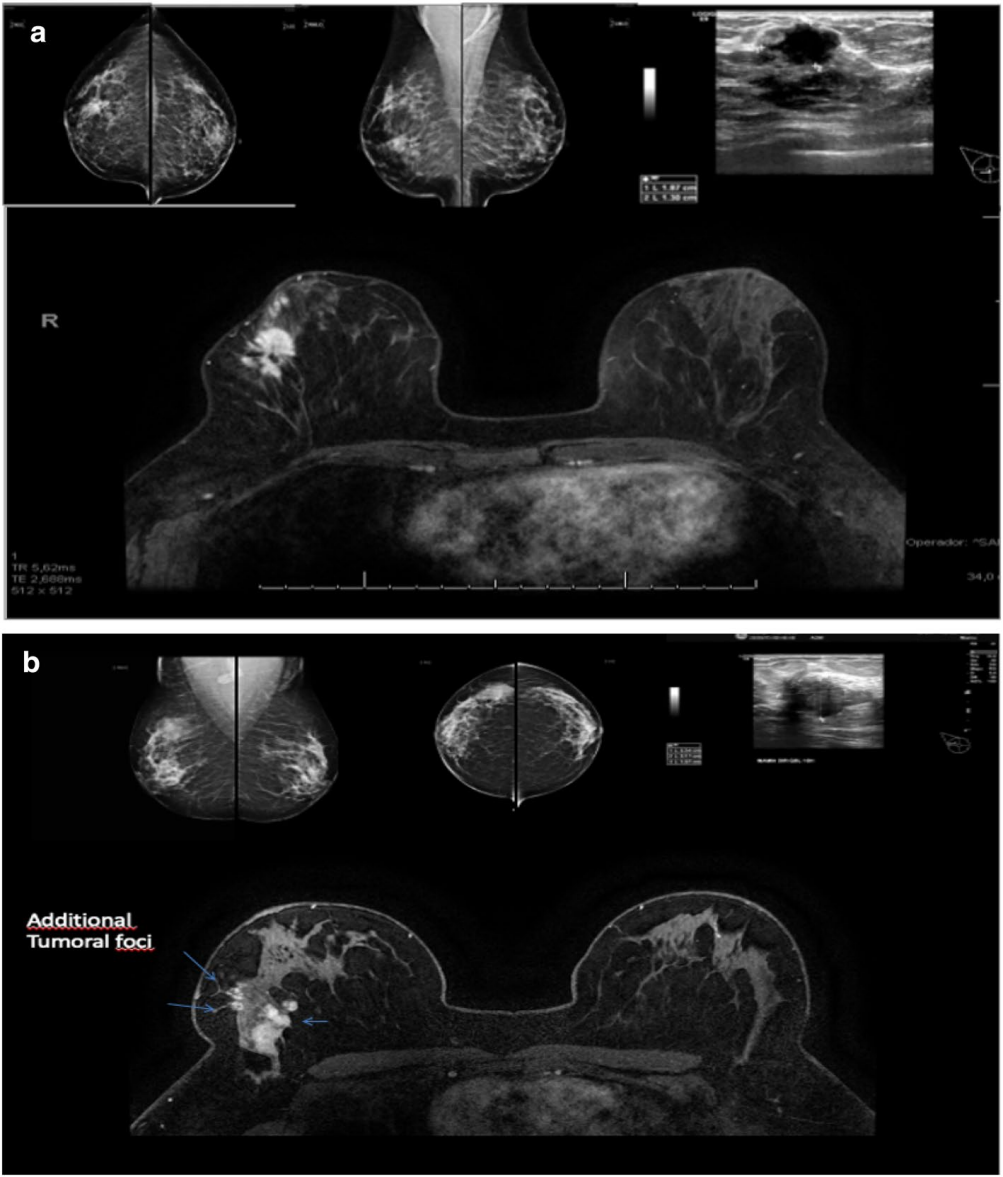
MRI additional findings. **a** Mastectomy due to a tumor 50% larger than evaluated by mammography and/or ultrasound and the breast still allows a conservative surgery. Mammography report (MMG): Hyperdense, irregular, and spiculated nodule, associated with tenuous amorphous calcifications, located in the lower outer quadrant (LOQ) of the right breast, measuring 1.8 × 2.8 cm. Corresponds to the irregular nodule in ultrasound are situated in the LOQ of the right breast, 1.9 × 1.3 × 1.3 cm. MRI report: Irregular nodule with spiculated margin, no signs of cutaneous involvement, located in the middle third of the LOQ of the right breast. Measures 3.0 × 3.0 × 2.0 cm. **b** Multicentricity tumor in MRI exam undetected by other methods. MMG report: focal asymmetry associated with round calcifications located in the right breast upper outer quadrant (UOQ), in agreement with an irregular nodule characterized at ultrasound in the right breast UOQ with 2.7 × 2.5 × 2.1 cm. The MRI report showed an irregular nodule with heterogeneous internal enhancement and progressive kinetic curve, placed in the posterior third of the UOQ of the right breast, measuring 4 × 3.4 × 2.7 cm. Associated with a focal clumped type enhancement with a progressive kinetic curve, with an extension of 3.1 cm anterior to it, which together measure 5.6 cm

All patients included in this trial were candidates for breast-conserving surgery (lumpectomy) based on triple assessment breast evaluation. In the intervention group according to MRI findings, the surgical management could change from lumpectomy to mastectomy. The Lumpectomy was considered when conservative breast surgery was performed according to the initial surgical plan or when small changes to the initial surgical plan were considered irrelevant. Mastectomy was performed when breast conservation was not possible due to MRI findings: (a) the breast does not support a conservative surgery due to aesthetical reasons, and the tumor was 50% larger than evaluated by mammography and ultrasound (Fig. 1a); or (b) multicentricity previously undetected by other imaging methods (Fig. 1b). Breast reconstruction techniques were performed if necessary.

The two criteria, 50% threshold or/and multifocality and breast patient volume evaluation not supporting a breast conservative surgery, were defined in a consensus meeting at our institution with input from members of our multidisciplinary team. According to the surgeon’s judgment, the surgery was modified to skin-sparing or nipple-sparing mastectomy after assessing the MRI and the distance between the lesions and the areolar complex. At least three surgeons were needed to confirm the decision to modify the surgical approach to mastectomy during the outpatient clinic visit. No mastectomy was performed if there was no unanimous agreement.

When necessary, preoperative localization was performed in all conservative breast surgeries using radio-guided occult lesion localization or wire localization (Kopans needle). Sentinel lymph node biopsy was performed using radio colloid or blue dye techniques.

Lumpectomy was considered adequate when clear margins were achieved on histopathological exams. On the other hand, if clear margins were not achieved, these patients were submitted to a new surgical procedure, either re-excision or conversion to mastectomy.

All lumpectomies and sentinel node biopsies were sent for intraoperative frozen-section analysis. Axillary lymph node dissection was recommended for patients with lymph nodal macro-metastases in more than two sentinel lymph nodes according to Z011 criteria [10]. After that, participants were submitted to postoperative histopathological processing. A clear margin was defined as a tumor not touching the inked border for invasive breast carcinomas. For ductal carcinoma in situ, 2 mm margins were considered clear on the final histopathological exam according to National Comprehensive Cancer Network guidelines [11].

The surgical management modification was correct if the histopathology showed that the index lesion’s size was at least 50% larger, as measured by MRI, or if there were multifocal or multicentric lesions. Postoperative adjuvant treatment was conducted according to local treatment guidelines [12]. Patient follow-up was every 6 months with a clinical exam and annual mammography. Ultrasound was not routinely done. A breast MRI was performed only to control BIRADS 3 lesions detected by the previous MRI. [11]

### Outcomes

The primary outcome of the BREAST-MRI trial was local relapse-free survival (LRFS). All the locoregional recurrences were confirmed by biopsy. The secondary outcomes were overall survival (OS), the proportion of patients whose surgical approach was modified to mastectomy, and the reoperation rate.

The LRFS was defined as the length of time after primary treatment for breast cancer that the patient survives without any locoregional signs or symptoms. All the locoregional recurrences were confirmed by biopsy. The OS has considered the length of time after primary treatment for breast cancer that the patient was still alive. The proportion of patients whose surgical approach was modified to mastectomy was the percentage of patients who have changed the surgical management due to MRI findings before surgery. The reoperation rate is the percentage of new surgeries to achieve clear margins until 6 months from the first surgery.

### Sample size

For sample size calculation, we estimated a difference in the local recurrence rate of 7% between conservative surgery and mastectomy as reported in the literature in twenty years of follow-up [1, 2]. A type-1 error of 5% (alpha) and type-2 error of 90% (beta) were assumed. We estimated a sample size of 518 participants, allowing for a loss of follow-up of 20%.

Since by the time our trial was activated, there was no available evidence to estimate the impact of MRI on local recurrence after breast-conserving surgery, we used the mastectomy rate for sample size calculation. We hypothesized that MRI would increase the mastectomy rate due to the finding of additional foci and decrease local relapse. We used that hypothesis as a surrogate for our sample size calculation.

### Randomization and allocation concealment procedures

To ensure homogeneity between the groups, randomization was matched and conducted by an independent statistician who did not know the participants, using a 1:1 ratio and stratified according to mammographic density (A, B, C e D). The sequence random generation was maintained in sequentially numbered, opaque, and sealed envelopes. One researcher informed the participant by phone to which group they were allocated before the schedule of the MRI exam.

### Statistical analysis

#### Variable and outcome analyses

The analysis of continuous variables was performed using measures of central tendency (including mean and median) and measures of dispersion. The Kolmogorov–Smirnov and Shapiro–Wilk tests were applied to assess data distribution characteristics. We used the Chi-square test or Fisher's exact test to compare outcomes with categorical variables. If it was non-normally distributed data, we used the nonparametric Mann–Whitney U test. The risk ratio was used to estimate the effect size for dichotomous outcomes effect size for dichotomous outcomes.

The time-to-local recurrence and OS were analyzed using the Kaplan-Meyer survival function with a stratified log-rank test and HRs estimated via a stratified Cox regression model to compare treatment groups. The follow-up losses and deaths were censored. The data were analyzed using the SPSS v 20.0 program. For all tests, a significance level of 5% was considered. The analyses were performed in the intention to treat the population, which included all randomized patients.

## Results

**Fig. 2:**
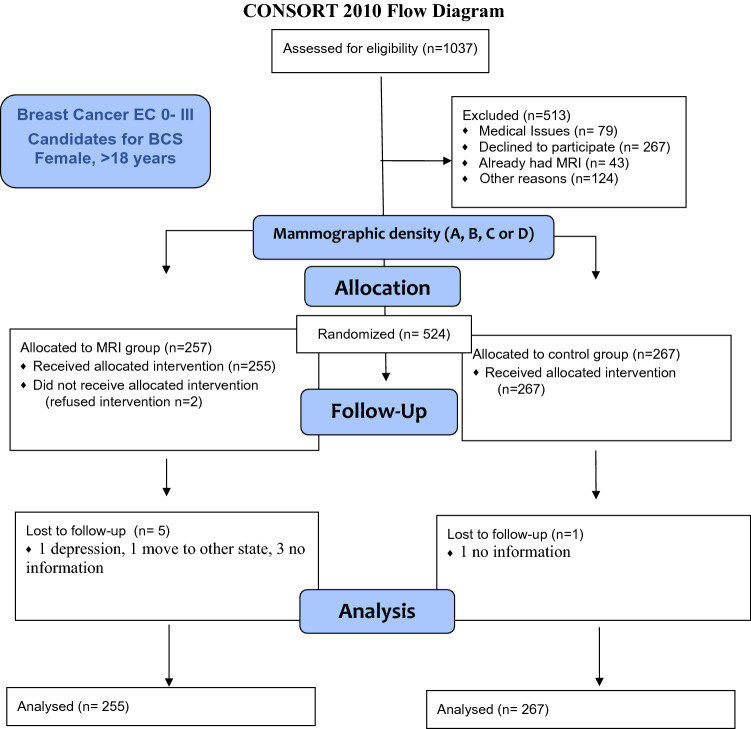
Flow chart of Breast MRI trial

Overall, 1037 patients were eligible for the trial; from those, 524 provided written consent and were included in the BREAST-MRI trial: 255 in the MRI group and 267 in the control group. Further, two participants refused to perform breast MRI and were withdrawn. The CONSORT flowchart of included participants is presented in Fig. 2.

The baseline characteristics were similar between groups (Table 1), except for adjuvant chemotherapy. An exploratory analysis considering only invasive carcinoma showed no difference in mean tumor size, being 2.2 cm (± 1.3) in the intervention group and 1.9 cm (± 1.08) in the control group (*p* = 0.06).

**Table 1:** Baseline characteristics

	MRI group (*N* = 255)	Control group (*N* = 267)	*P*
Age (median)	56.9	57.1	0.79
Body mass index (kg/m^2^)	29.1	29.4	0.3
Nulliparity			0.55
Yes	26 (10.2%)	30 (11.2%)	
No	228 (89.4%)	237 (88.8%)	
Missing information	1	0	
Menopausal status			0.35
Premenopausal	88 (34.5%)	82 (30.7%)	
Postmenopausal	167 (65.5%)	185 (69.3%)	
HRT			0.10
No	230 (90.2%)	241 (90.3%)	
More than five years	3 (1.2%)	9 (3.4%)	
Less than five years	19 (7.5%)	17 (6.4%)	
Mammary density			0.73
A	15 (5.9%)	16 (6.0%)	
B	109 (42.7%)	119 (44.6%)	
C	115 (45.1%)	121 (45.3%)	
D	16 (6.3%)	11 (4.1%)	
Clinical Stage (Initial)			0.34
0	34 (13.3%)	40 (15%)	
I	119 (46.7%)	124 (46.4%)	
II	99 (38.8%)	103 (38.6%)	
III	3 (1.2%)	0	
Histological classification			0.94
IDC	194 (76.1%)	207 (77.5%)	
ILC	13 (5.1%)	12 (4.5%)	
DCIS	31 (12.2%)	32 (12%)	
Others	17 (6.7%)	16 (6.0%)	
Time to surgery*	72.7 (7–157)	65.1 (7–155)	0.001
Immunohistochemical			0.95
HR +	206 (81.1%)	211 (79.6%)	
Her 2	6 (2.4%)	7 (2.6%)	
Her2/HR+	24 (9.4%)	25 (9.4%)	
Triple negative	18 (7.1%)	22 (8.3%)	
Missing	1	2	
Chemotherapy			0.006
Yes	133 (52.2%)	107 (40.1%)	
No	122 (47.8%)	160 (59.9%)	
Radiotherapy			1.00
Yes	242 (94.9%)	254 (94.7%)	
No	13 (5.1%)	13 (4.9%)	
Hormone therapy			1.00
Yes	222 (87.1%)	232 (86.9%)	
No	33 (12.9%)	35 (13.1%)	
Pathological stage (Final)			0.26
0	28 (12.9%)	34 (14.9%)	
I	97 (44.7%)	108 (47.4%)	
II	89 (41.0%)	86 (37.7%)	
III	3 (1.4%)	0 (0%)	
Follow-up (years)	6,1	6.2	0.99
Status			0.21
Alive	239 (93.7%)	251 (94%)	
Local recurrence	2 (0.7%	3 (1.1%)	
Distant recurrence	4 (1.6%)	5 (1.9%)	
Death (all causes)	12 (4.7%)	10 (3.7%)	

*IDC* invasive ductal carcinoma; *ILC* invasive lobular carcinoma; *HRT* hormone replace therapy; *HR* hormonal receptor

*One patient from the MRI group was excluded from the time analyses due to comorbidity (patient undergone surgery because of a Schwannoma with intracranial hypertension before breast surgery)

The mean time from randomization to surgery was different between groups, 72.7 days (± 32.1) in the MRI group and 65.1 days (± 36.4) in the group control group (*p* = 0.001). Preoperative localization was performed in all conservative breast surgeries when necessary: the tumor was palpable in 147 cases (29.4%), wire localization with Kopans needle in 57 (11.4%), and radio-guided occult lesion localization in 296 cases (59.2%).

The MRI group had 46 additional biopsies in 44 patients versus 22 additional biopsies in 21 patients in the control group (*p* 0.005). Of 46 additional biopsies in the MRI group, 25 were motivated by MRI, 13 by mammography, and 8 by USG versus 14 by mammography and 8 by USG in the control group. Eleven out of 65 (16.9%) additional biopsies in ipsilateral breasts were confirmed to be invasive carcinoma (10 in the MRI group and 1 in the control group), 2 DCIS (1 in the MRI group and 1 in the control group), 1 lobular in situ carcinoma in the MRI group, 8 atypical lesions (2 in the MRI group and 6 in the control group), 6 discordant benign (5 in the MRI group and 1 in the control group), and 37 concordant benign (26 in the MRI group and 11 in the control group)(Table2).

**Table 2:** Additional findings by exam and biopsies performed in the pre-surgical planning

	MRI	Control group	p
Number of Additional biopsies in pre-surgical planning	46 (44 patients)	22 (21patients)	0.005
MRI	25		
MMG	13	14	
USG	8	8	
Result of additional biopsies			
Invasive carcinoma	10	1	
DCIS	1	1	
LCIS	1	0	
Atypical lesions	2	6	
Discordant benign	5	1	
Concordant Benign	26	11	

*MRI* Magnetic Resonance Image; *MMG* Mammography; *USG* ultrasound; *DCIS* ductal in situ carcinoma; *LCIS* lobular in situ carcinoma

### Local recurrence-free survival

**Fig. 3:**
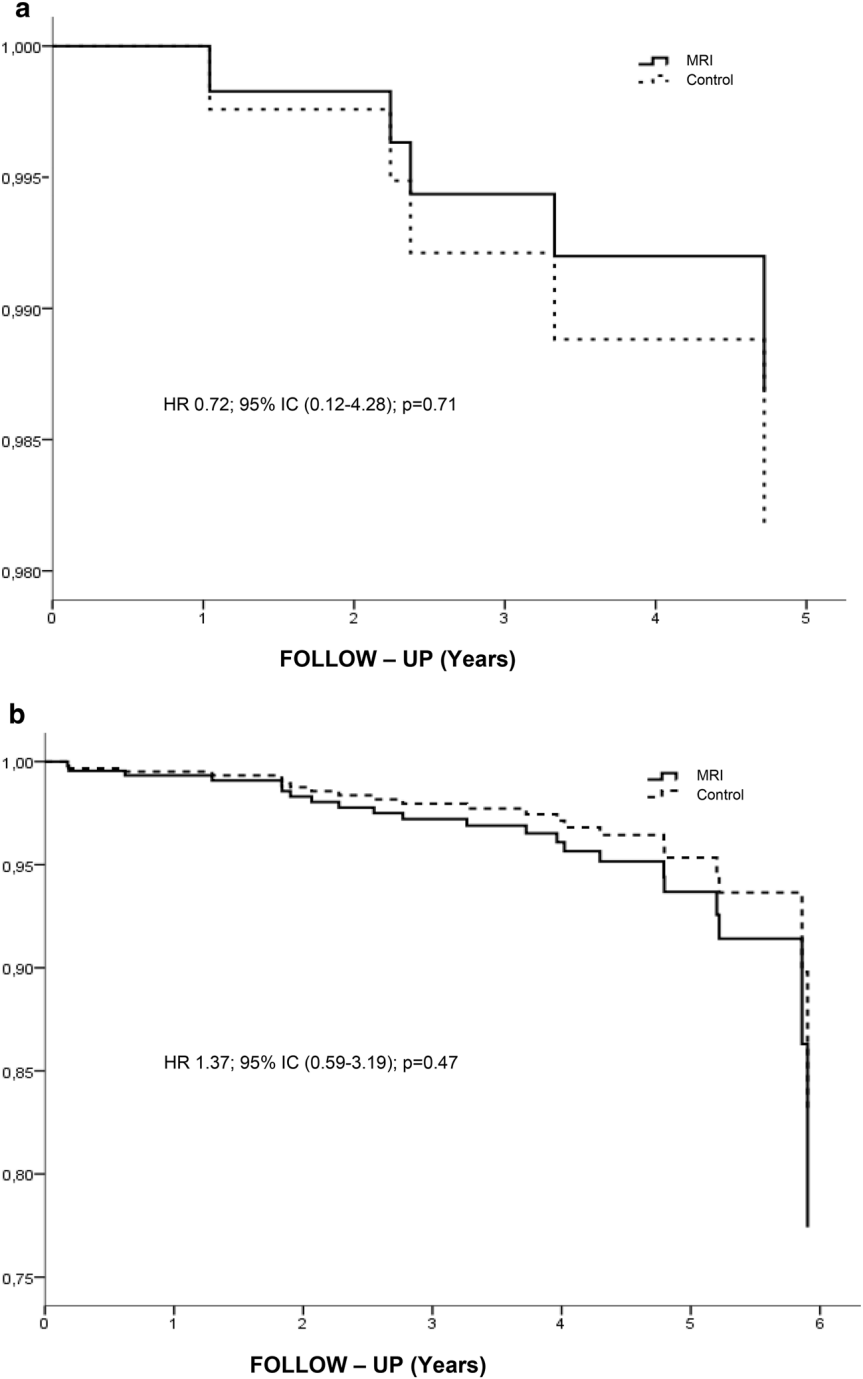
Local recurrence-free survival and Overall survival. **a** Local recurrence-free survival. **b** Overall survival

After a median follow-up time of six years, there were two (1.6%) local recurrences in the MRI group versus three (2.2%) in the control group. The 5.9-year local recurrence-free survival was 99.2% in the MRI group *versus* 98.9% in the control group (HR = 0.72; 95%CI 0.12—4.28; log-rank test,* P* = 0.7, Fig. 3a).

### Overall survival

After a median follow-up time of 5.8 years, 12 deaths were observed in the MRI group versus ten patients in the control group. The OS was 95.3% in the MRI group *versus* 96.3% in the control group (HR = 1.37 95%CI 0.59–3.19; log-rank test, *P* = 0.8, Fig. 3b).

### Surgical approach modified to mastectomy

Overall, 21 (8.3%) patients had their initial surgical procedure changed to mastectomy due to MRI findings in the ipsilateral breast according to our management modification criteria. Eight patients had lesions > 50% of the original size; 8 had multicentric/multifocal tumors, and 5 had both criteria for change (> 50% and multifocal) and breasts that did not support conservative surgery. Five out of 21 patients, the patient changed their surgery incorrectly, and there is no agreement with the pathology (1 participant from 50% larger criteria, 2 participants multifocal criteria and 2 participants from 50% larger and multifocal criteria) (Table 3). Of 21 patients whose surgical management was changed in the MRI group, nine were submitted to additional biopsies guided by second-look ultrasound with the following results: five invasive carcinomas, one ductal carcinoma in situ, and three discordant benign. In the control group, only one (0.4%) patient had undergone an MT due to aesthetic reasons—conversion to MT was made intraoperatively after a wide lumpectomy was needed to achieve clear margins, and pre-planned mammoplasty was not suitable anymore. (Table 4).

**Table 3:** Patients’ characteristics and image findings from the 21 patients in MRI group that had surgical management changed to mastectomy due to MRI findings

Patient ID	Age (years)	BD	MG	US	MRI	Final histopathology	Criteria for change management	Was the change appropriate?
Case 1	74	B	Architectural distortion 2.2 cm	Irregular mass 2.2 cm	Lesion 1: NME clumped 4.1 cm Lesion 2: NME focal another quadrant 0.7 cm	Foci 1: ILC 3.5 cm with DCIS 10% Foci 2: ILC 3 mm in another quadrant	> 50% larger and Multicentric/ Multifocal	Yes
Case 2	65	C	Irregular mass 5.6 cm	Irregular mass 5.4 cm	Lesion 1: Irregular mass 6.5 cm Lesion 2: NME linear 3.7 cm	Papillary invasive carcinoma 5.0 cm + IDC 0.7 cm with DCIS 5%	Multicentric/ Multifocal	Yes
Case 3	50	C	Architectural distortion 2.4 cm	Irregular mass 1.7 cm	NME segmental 5.6 cm	IDC 3.0 cm with DCIS 1.2 cm (40%) multifocal	> 50% larger	Yes
Case 4	46	C	Irregular mass 1.8 cm	Irregular mass 1.8 cm	Lesion 1: Irregular mass 1.5 cm Lesion 2: mass 1.1 cm same quadrant Lesion 3: mass another quadrant 1.0 cm	Foci 1: IDC 3.0 cm with DCIS 5% Foci 2: benign Foci 3: IDC 2.5 cm with DCIS 5%	Multicentric/ Multifocal	Yes
Case 5	51	B	Irregular mass 3.0 cm	Irregular mass 3.4 cm	Lesion 1: irregular mass 3.1 cm Lesion 2: mass 0.6 cm same quadrant	Foci 1: IDC 3.8 cm Foci 2: IDC 0.6 cm	Multicentric/ Multifocal	Yes
Case 6	66	B	Irregular mass 1.9 cm	Irregular mass 1.4 cm	Lesion 1: Irregular mass 2.8 cm Lesion 2: mass 0.5 cm same quadrant Lesion 3: mass another quadrant 1.4 cm	Foci 1: IDC 2.1 cm Foci 2: IDC 4 mm with DCIS 70% Foci 3: IDC 1.2 cm	Multicentric/ Multifocal	Yes
Case 7	53	C	Architectural distortion 0.9 cm	Irregular mass 0.9 cm	Lesion 1: irregular mass 2.0 cm Lesion 2: mass 1.6 cm same quadrant	Foci 1 and 2: ILC 3.1 cm with CLIS 10%	> 50% larger and Multicentric/ Multifocal	Yes
Case 8	44	C	Calcifications 0.7 cm	No lesion	Lesion 1: NME linear 0.7 cm Lesion 2: NME segmental 3.1 cm lesion 3: mass 1.0 another quadrant	Foci 1 and 2: IDC 1.6 cm + DCIS 60%, multifocal Foci 3: papilloma	> 50% larger and Multicentric/ Multifocal	Yes
Case 9	63	B	Irregular mass 2.0 cm	Irregular mass 2.2 cm	Lesion 1: irregular mass 5.2 cm Lesion 2: mass 0.8 cm same quadrant	Foci 1: IDC 3.6 cm Foci 2: benign	> = 50% larger	Yes *
Case 10	54	C	Architectural distortion 6.5 cm	Irregular mass 1.4 cm	Irregular mass 10 cm	IDC 4.5 cm	> = 50% larger	No
Case 11	61	C	Irregular mass 2.4 cm	Irregular mass 2.6 cm	Lesion 1: irregular mass 2.9 cm Lesion 2: mass another quadrant 0.6 cm	Foci 1: Papillary invasive carcinoma 2.7 cm Foci 2: Papillary invasive carcinoma 0.3 cm	Multicentric/ Multifocal	Yes
Case 12	64	B	Irregular mass 1.5 cm	Irregular mass 1.5 cm	Lesion 1: irregular mass 2.9 cm Lesion 2: mass same quadrant 1.2 cm	IDC 3.3 cm associated with DCIS 5%	> 50% larger	Yes
Case 13	40	C	Architectural distortion 2.5 cm	Irregular mass 2.7 cm	Lesion 1: irregular mass 2.2 cm Lesion 2: mass another quadrant 0.9 cm	Foci 1: IDC 2.4 cm + DCIS 10% Foci 2: fibroadenoma	Multicentric/ Multifocal	No
Case 14	73	C	Calcifications 2.8 cm	No lesion	No index lesion Lesion 2: mass another quadrant 0.6 cm	Foci 1 and 2: IDC 0.5 cm + IDC 0.3 cm (same quadrant) Foci 3: IDC 0.2 cm with DCIS 80%	Multicentric/ Multifocal	Yes
Case 15	66	B	Irregular mass 2.0 cm	Irregular mass 3.0 cm	Lesion 1: irregular mass 2.2 cm Lesion 2: NME clumped 5.0 cm	IDC 6.9 cm	> 50% larger	Yes
Case 16	45	C	Irregular mass 2.2 cm	Irregular mass 2.2 cm	Lesion 1: irregular mass 2.7 cm Lesion 2: mass same quadrant 5.2 cm	IDC 9.0 cm	> 50% larger	Yes
Case 17	36	D	Irregular mass 2.2 cm + calcifications 1.1 cm	Irregular mass 2.2 cm + architectural distortion 1.1 cm	Irregular mass 2.5 cm associated with 3 additional irregular masses same quadrant, 8.1 cm total	Mucinous invasive carcinoma 8.0 cm	> 50% larger	Yes
Case 18	58	C	Architectural distortion 1.8 cm	Irregular mass 1.5 cm	NME segmental 5.0 cm	ILC 9.7 cm	> 50% larger	Yes
Case 19	50	B	Calcifications 1.2 cm	No lesion	Lesion 1: NME segmental 4.5 cm Lesion 2: NME segmental another quadrant	DCIS 2.4 cm	> 50% larger and Multicentric/ Multifocal	No
Case 20	54	B	Calcifications 7.7 cm	Irregular Mass 1.0 cm	Lesion 1: NME segmental 5.0 cm Lesion 2: mass 2.6 cm another quadrant	Foci 1: IDC 1.3 cm + DCIS 5% Foci 2: benign	Multicentric/ Multifocal	No
Case 21	70	A	Irregular mass 0.8 cm + calcifications 4.3 cm	Irregular Mass 0.8 cm	Lesion 1: irregular mass 1.3 cm Lesion 2: NME segmental 10 cm	IDC 1.7 cm with DCIS, multifocal	> 50% larger and Multicentric/ Multifocal	No

*BD* breast density; *MG* mammogram; *US* ultrasound; MRI magnetic resonance image; *NME* non-mass enhancement; *MD* mammographic density; IDC invasive ductal carcinoma; *DCIS* ductal carcinoma in situ

*We considered the surgical planning change as appropriate due to the histopathological exam showed a tumor more than 50% from MG and the patient’s breast does not allow a conservative surgery with this size tumor

**Table 4:** Breast surgical management and repeated operation rates

	MRI Group (255 patients)	Control Group (267 patients)	Total	*P*
Initial Surgery				< 0.001
Lumpectomy	234 (91.8%)	266 (99.6%)	458 (87.7%)	
Mastectomy	21 (8.3%)	1 (0.4%)	22 (4.2%)	
Further Surgery				1.0
No	233 (91.4%)	244 (91.6%)	477 (91.4%)	
Yes	22 (8.6%)	23 (8.6%)	45 (8.6%)	
Re-excision	17(6.7%)	17 (6.4%)	34 (6.5%)	
Mastectomy	5 (2.3%)	6 (2.2%)	11 (2.1%)	

In an exploratory analysis to assess the potential role of MRI in dense breasts, we compared patients with dense breasts that had their surgery changed to mastectomy versus patients without dense breasts that had their surgery changed to mastectomy. In the ipsilateral breast, nine procedures in A/B breasts and 13 procedures in C/D breasts were correctly modified. There was no difference between these groups (RR = 0.79; 95% CI 0.35—1.81; *p* = 0.65).

### Reoperation rate

No difference was found in reoperation rates, 22 (8.7%) in the MRI group versus 23 (8.7%) in the control group (RR = 1.002; 95%CI 0.57–1.75;* p* = 0.85).

Re-excisions were necessary for 17 (6.7%) participants in the MRI group and 17 (6.4%) participants in the control group. Mastectomies were necessary for 5 (2%) participants in the MRI group and 6 (2.3%) in the control group (Table 4). The final mastectomies rates were 26 (10.2%) in MRI groups versus 7 (2.6%) in the control group (RR 3.889; 95%CI (1.71—8.8; *p* = 0.000).

## Discussion

Our results show that preoperative breast MRI did not change the local recurrence and overall survival rates in breast-conserving surgery candidates. Additionally, preoperative breast MRI increased the mastectomy rates and did not reduce reoperation rates.

Only a few studies have examined the long-term outcome effects of preoperative MRI. A previous systematic review that included 3169 patients with published studies until 2012 demonstrated that 8-year disease-free survival did not differ between the MRI (89.0%) and no-MRI (93.0%) groups (*p* = 0.37) [13]. A larger retrospective study involving 1030 patients with invasive cancer found that local recurrence rates after 8 years with and without MRI were 4.2% vs. 7.3% (*p* = 0.28), and for 366 DCIS patients with and without MRI, the IBTR was 3.6% vs. 10.9% (*p* = 0.06).[14].

Despite local recurrence-free survival early data with 6-year follow-up in our trial, it corroborates with those data. These results may be due to the benefit of radiotherapy in treating undetected findings in the control group and due to the benefit of adjuvant systemic treatment as described in the multivariate analysis of this cohort study where radiotherapy and endocrine therapy were independent factors to prevent local recurrence with a benefit of 86% varying 93% to 70% according to the confidence interval for both treatments. In this cohort, there was an increase in the percentage of adjuvant chemotherapy in the MRI group, explained by the largest median tumor in this group, although this fact did not have an impact on the local recurrence rate (RR 0.9; 95%IC 0.49–1.59) [14]. Based on the observed rate of LR in our study being significantly lower than predicted, the study lacked the power to identify a significant difference in rates of LR or survival.

The criteria for modifying the surgical management based on additional findings in MRI are different in prospective and retrospective studies published until now. There is an increase in mastectomy rates in most previously published studies when preoperative staging with breast MRI is performed with percentages from 7 to 20% [8, 15]. Our study confirmed these findings with an increase of 8.3% in mastectomy rates. The MRI as a preoperative evaluation in breast cancer patients increases the risk of mastectomy by 3.8 times more compared to patients who had this evaluation with clinical examination, bilateral mammogram, and ultrasound. A post-hoc analysis to evaluate our study's power to answer this question showed 99% of power.

The core issue about the MRI exam to pre-operatory staging is the unnecessary mastectomies, due to false positive findings. Three out of five clinical trials performed the correlation between breast MRI and histopathological findings [16–18], and two described the false positive rates. The false positive rate in the POMB trial [19] was 9% (2 out of 22 mastectomies) and 38% in the COMICE trial (55 out of 144 mastectomies). [18].”All these trials recommended for multifocal/multicentric lesions performed additional exams when additional lesions were detected using a second-look ultrasound and biopsy guided by US or MRI according to these findings. Therefore, is not possible to identify the number of participants who have undergone mastectomy without investigating the additional foci which could affect the number of overtreatments. In our trial, the false positive rate was 23.8%. In our database, only 9 out of 13 (62%) of multifocal/multicentric findings were submitted to an additional biopsy by USG, due to MRI -biopsy is not available in our hospital. Of 23% (5 participants) of false positive cases, 4 were not performed additional biopsies and one had a benign discordant biopsy.

In this trial, no difference in reducing reoperation rates between the MRI and control groups was observed, which remains controversial in the literature. Although retrospective studies showed robust evidence in reducing repeated surgeries [16], randomized studies have conflicting results on this subject [15, 16, 18–20]. Retrospective studies have a higher risk of bias, increasing the effect size, and leading to a spurious association [21]. Of the five randomized trials published on this subject, three did not show differences in reoperation with preoperative MRI [16, 18, 22], one found an increased number of additional procedures [20], and one reported a reduced number of additional surgeries [19]. POMB trial, the only one with a reduction of re-operative rates, was a prospective trial that included 440 young patients [19]. It found that the breast reoperation rate was significantly lower in the MRI group: 11 of 220 (5%) versus 33 of 220 (15%) in the control group (*p* = 0.001). Therefore, the study with the largest sample size including 1623 participants did not show this benefit with 19% of re-excision rates in both groups (Table5) [18].

**Table 5:** Clinical trial summaries available until 2023

Trial	Stage	*n*	Re-excision rates		*p* value	Mastectomy Rates (final surgery)		*p* value
			MRI (%)	Control (%)		MRI (%)	Control (%)	
COMICE,2010	IC and DCIS*	1625	10	11	0.77	13	9	
Monet, 2011	Benign disease IC and DCIS**	418	34	12	0.008	11	14	0.49
POMB, 2014	IC and DCIS***	440	5	15	0.001	20	10	0.024
Bruck et al., 2018	I	100	14	24	0.202	12	4	0.140
IRCIS, 2019	DCIS	352	20	27	0.68	18	17	0.93
BREAST-MRI	0—III	524	8.3	8.6	1.0	10.6	2.6	0.000

IC invasive carcinoma; DCIS: Ductal in situ carcinoma

*The author not described the stage tumor

**The author included B3, B4 and B5 at breast exam. In the MRI group were 39 IC and 39 DCIS, and 38 IC and 41 DCIS

***The author included neoadjuvant treatment and did not describe the stage tumor

There are five randomized studies evaluating the impact of conservative surgery on surgical planning, all of them had objective final mastectomy rates and repeat surgery [16, 18–20]. Most of them included invasive and DCIS [18–20], one only Stage I tumor [15], and one only DCIS [16]. The number of participants was wide in trials. The COMICE trial included 1623 participants, of which 1466 invasive carcinomas [18]. The MONET trial included 463 participants with BIRADS 3–5 lesions; of which 299 were benign lesions and 81 were invasive breast cancer and 82 DCIS [20]; the POMB trial included 440 participants with invasive and in situ (does not mention the number in each arm). Moreover, this trial included 54 participants undergoing neoadjuvant chemotherapy [19]. Bruck et al. included 143 participants with stage 1 tumors [15], and IRCIS included only 352 participants with DCIS tumors [16]. The criteria for conversion to mastectomy was tumor-to-breast volume ratio in one study [15], MRI lesion more than 1 cm longer than triple assessment [19], and more than 3 cm or multifocality [16] and not mentioned in 2 studies [18, 20]. Regarding sample size calculation, four out of five described it [16, 18–20]. The Monet trial did not achieve recruitment; the study included only 35% of invasive and in situ diseases which could underpower the study [20]. The other studies achieve the recruitment number using the following percentages of change planned surgical management by MRI to perform the sample size calculation 26% at the POMB trial [19], a 50% of relative reduction in the IRCIS trial [16], and a reduction in 5% of re-operation rates [18]. In our trial, we included a total of 524 invasive and DCIS tumors based on the assumption of a difference of 7% local recurrence rate between conservative and mastectomy with an objective to evaluate if the MRI could avoid a local recurrence during follow-up (Table 5).

The design of our trial has two novel strengths. To the best of our knowledge, the BREAST-MRI trial is the first to use randomized stratification based on mammographic density to evaluate the performance of breast MRI in different subgroups. Furthermore, the selection of a measurable threshold to change surgical indication contributes to the literature by adding an objective criterion to the subjective choice of individual surgeons. Occasionally, the definition of 50% larger may favor breast MRI performance without having any clinical relevance in very small tumors. However, the median tumor size for DCIS and invasive carcinoma in breast MRI was 2.9 cm with an interquartile range of 1.5 cm, and the tumor-to-breast volume ratio was always taken into account when making a decision. So, we believe that there was a real impact when it comes to planning conservative surgeries. As potential strengths of our study, we collaborated with breast radiologists with more than five years of experience that interpreted all imaging exams, and we followed patients rigorously, so there were only four losses on follow-up.

This trial's main limitations were the unbalance between groups regarding the use of adjuvant chemotherapy, the lack of the allocation concealment procedure, and unblinding evaluators' outcomes and low rate of local recurrence. Nevertheless, the clinical stages were similar between groups; the protocol for chemotherapy treatment in our institution is based on clinical features, including a tumor size of more than 2.0 cm, which could have led to an increase in this treatment in the MRI group. Despite the lack of allocation concealment procedure, only two patients refused to perform MRI in the intervention arm, and this is very unlikely to impact the outcome. Regarding unblinding evaluators’ outcomes, the mastectomy rates are likely to be influenced, and trying to avoid the detection bias we had 3 senior breast surgeons to perform this decision. The other outcomes (all local recurrences were confirmed by biopsy, death, and reoperation rate) are very objective and unlikely to increase the detection bias. Another issue is the time from randomization to surgery; there is a statistical difference between groups showing ten days more in the MRI group, which is explained by the need for additional biopsy. In both groups, time took at least two months to undergo surgery. This prolonged time may happen due to our institution's characteristic, the biggest tertiary hospital in Brazil, with a massive number of patients with low-quality imaging studies before the referral; this incurs due to the necessity to repeat most of the exams after their first visit at ICESP. The low rate of local recurrence probably occurred due to the sample size calculation was based on a study with 20y-follow-up, to achieve 80% of power in this period, we need almost 5800 participants [2], 2900 participants *per* arm which are difficult to have in a single-center study. Our group will publish updated results when we reach 20 years of follow-up to evaluate this outcome.

As for implications in clinical practice, MRI is widely used in preoperative breast cancer patients leading to higher mastectomy rates with no strong evidence that it could avoid a local recurrence. Our trial has an increase of almost 8% in surgical change to mastectomies and, our early results showed that the use of breast MRI did not impact oncological outcomes. In daily practice, its use should be based on shared decisions with breast cancer patients.

Regarding future research, in the era of treatment de-escalation and based on the scientific GAP about local recurrence protection, we believe that the publication of the interim analysis is of essential importance to guide other groups and can also be used as a basis for multicentric studies since this the first trial with local recurrence-free survival as a primary outcome. We also believe that further analysis to assess the cost-effectiveness of breast MRI according to the number of unnecessary biopsies or surgeries must be planned.

## Conclusion

This randomized controlled trial supports that preoperative breast MRI may increase the mastectomy rates and does not routinely change local relapse-free survival, overall survival, and reoperation rates in early-stage breast cancer in this interim analysis, and its use should be based on shared decision-making with patients.

## Data Availability

The data is available with the author in the redcap platform if anyone requires.
